# Retraction: Fumagillin regulates stemness and malignancies in cancer stem-like cells derived from liver cancer via targeting to MetAP-2

**DOI:** 10.1371/journal.pone.0300802

**Published:** 2024-03-26

**Authors:** The PLOS ONE Editors

Following the publication of this article [1], concerns were raised regarding results presented in Figs 1, 2, 5, 7, and 8. Specifically, the following panels appear highly similar but were reported as representing different experimental results:

- The Fig 1A SNU-449 CSCs Mock panel of [1] and the Fig 4C LV-HOTAIR panel of [2, retracted in 3], which was previously published under a CC-BY 4.0 license by a different author group.
- Fig 1B Huh-7 CSCs Oct-4 lanes 1–3 and Fig 8E SNU-449 p53 when flipped horizontally.
- Fig 1B SUN-449 CSCs Oct-4 lanes 1–3 and Fig 8E Huh-7 p53 when flipped horizontally.
- Fig 2C β-actin and Fig 2D β-actin.
- The Fig 5B upper row (Huh-7 CSCs) Doxorubicin and Sorafenib panels.
- The Fig 7D SNU-449 CSCs Mock panel in [1] and the Fig 4C U87MG Vector panel in [4, retracted in 5] which was published after [1] by a different author group.

The authors provided data underlying the published results, but these data were not sufficient to resolve the above concerns.

The above concerns call into question the integrity and reliability of these data, and so the *PLOS ONE* Editors retract this article.

ZZ responded but expressed neither agreement nor disagreement with the editorial decision. KZ and JH either did not respond directly or could not be reached.
